# Novel Data Transformations for RNA-seq Differential Expression Analysis

**DOI:** 10.1038/s41598-019-41315-w

**Published:** 2019-03-18

**Authors:** Zeyu Zhang, Danyang Yu, Minseok Seo, Craig P. Hersh, Scott T. Weiss, Weiliang Qiu

**Affiliations:** 1 Department of Bioinformatics, School of Life Sciences and Technology, Tongji University, Shanghai, China; 2 Department of Information and Computing Science, College of Mathematics and Econometrics, Hunan University, Hunan, China; 3 Channing Division of Network Medicine, Brigham and Women’s Hospital/Harvard Medical School, Boston, USA

## Abstract

We propose eight data transformations (*r*, *r2*, *rv*, *rv2*, *l*, *l2*, *lv*, and *lv2*) for RNA-seq data analysis aiming to make the transformed sample mean to be representative of the distribution center since it is not always possible to transform count data to satisfy the normality assumption. Simulation studies showed that for data sets with small (e.g., nCases = nControls = 3) or large sample size (e.g., nCases = nControls = 100) limma based on data from the *l*, *l2*, and *r2* transformations performed better than limma based on data from the *voom* transformation in term of accuracy, FDR, and FNR. For datasets with moderate sample size (e.g., nCases = nControls = 30 or 50), limma with the *rv* and *rv2* transformations performed similarly to limma with the *voom* transformation. Real data analysis results are consistent with simulation analysis results: limma with the *r, l, r2*, and *l2* transformation performed better than limma with the *voom* transformation when sample sizes are small or large; limma with the *rv* and *rv2* transformations performed similarly to limma with the *voom* transformation when sample sizes are moderate. We also observed from our data analyses that for datasets with large sample size, the gene-selection via the Wilcoxon rank sum test (a non-parametric two sample test method) based on the raw data outperformed limma based on the transformed data.

## Introduction

With the rapid development of next-generation high throughput RNA sequencing technologies in recent years, genomics studies have seen tremendous advancement. RNA-seq technology is a type of next generation sequencing technology to estimate the expression levels of genes in whole-genome scale studies and has become the standard technology for the study of genomics^1,2^. RNA-seq technology can help identify new genes, with high-sensitivity, high signal-to-noise ratio and small sample requirements. Also, RNA-seq technology can measure read counts at exons, genes, or gene units. Therefore, RNA-seq sequencing technology has been widely used in many different research fields^3,4^.

RNA-seq data are usually represented by a matrix of counts showing the expression levels of mRNAs (rows) for a set of samples (columns) after processes such as adapter remove step, alignment step, and quantification step. For each sample, millions of reads can be measured by the RNA-seq technique^5^. According to the gene annotation and genome build, numbers of features might be different. Different pipelines, such as Cufflink pipeline, Hisat2-StringTie pipeline, and Star-FeatureCount pipeline could result in different properties of the count matrix. Two common properties are sparsity and skewness. Sparsity means that many counts in the count matrix are zero. Skewness means that the histogram of all counts in the count matrix is usually skewed. Skewness indicates that data transformation is required before applying linear regression analysis, which assumes data from normal distributions. Sparsity indicates that the log2 transformation, which is commonly used in gene microarray data, could not be directly applied to RNA-seq data analysis since log2(0) does not exist. It is still expensive to collect RNA-seq data for large sample size. Hence, existing RNA-seq datasets usually have small sample size. To address these two common properties, count distributions, such as Poisson, negative binomial, and inflated Poisson distributions, have been proposed to fit RNA-seq data^6,7^. Commonly used R Bioconductor packages that fit RNA-seq data using count distributions include edgeR^8,9^, DESeq^10^, and DESeq2^11^. These methods could borrow information across genes to increase the power of the tests for detecting genes differentially expressed between two conditions (e.g., cases versus controls).

The distributions of counts are not as statistical tractable as normal distributions^12^. Moreover, there are much fewer analytic tools for count distributions than there are for normal distributions in statistical analysis. Law *et al*.^12^ proposed the *voom* transformation to transform the count distribution to a distribution close to the normal distribution in RNA-seq data analysis and demonstrated that using *limma*^13^ with the *voom*-transformed count data performed comparable to count-based RNA-seq analysis methods, such as edgeR^8,9^, DESeq^10^, baySeq^14^ and DSS^15^.

The *voom* transformation is a sample-specific transformation, defined as log-counts per million (log-cpm):

$${y}_{gi}={\mathrm{log}}_{2}(\frac{{r}_{gi}+0.5}{{R}_{i}+1.0}\times {10}^{6})$$

where r_gi_ is the count of the g-th mRNA transcript for the i-th sample, R_i_ is the total counts (R_i_ = r_1i_ + r_2i_+ … +r_Gi_) for the i-th sample, g = 1, …, G, i = 1, …, n, G is the number of mRNA transcripts, and n is the number of samples.

**Figure 1:**
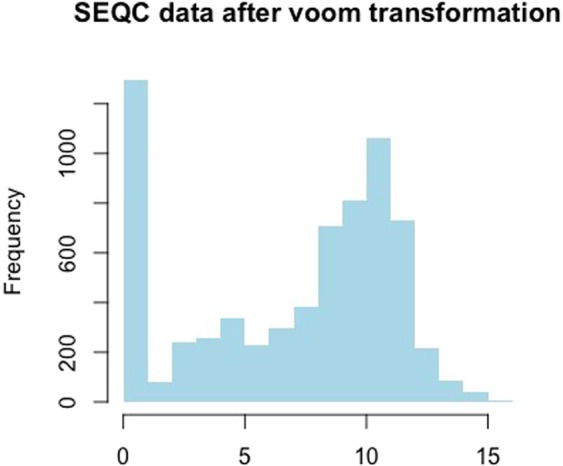
Histogram of the pooled SEQC RNA-seq data after the voom transformation. The histogram showed that the empirical distribution after the TMM scale normalization, quantile normalization and voom-transformation could still be far from a normal distribution.

The goal of the *voom* transformation is to make the empirical distribution of transformed RNA-seq data closer to a normal distribution so that the moderate t tests (*limma*) could be used. However, it is not always possible to transform count data to have a distribution closer to a normal distribution in real data analysis^16^. For example, for the SEQC data that was analyzed in real data analyses part of^12^, the empirical distribution (i.e., histogram) of the *voom* transformed data is still far from a normal distribution (Fig. 1). The histogram is based on the pooled data Ygi, g = 1, …, 92, i = 1, …, 8.

In this article, we proposed to relax the normality requirement for a data transformation. Most statistical models, such as two-sample t-test, focus on comparing the centers of the two distributions to check if two distributions are same or not. Sample means are usually used to represent distribution centers. However, for skewed distributions, sample means are not good to characterize the distribution centers. Instead, sample medians are usually used to characterize the centers of skewed distributions. However, sample medians do not have as tractable properties as sample means. For instance, it is hard to derive the distribution of sample median. In this article, we aim to transform the RNA-seq count data by minimizing the difference between sample mean and sample median so that the sample mean would be a good representative to the center of the transformed distribution. Hence, most existing statistical models based on sample means, e.g., *limma*, can be directly applied to analyze transformed RNA-seq data.

## Results

### Results for simulation studies

In this article, we proposed 8 data transformation methods to improve the *voom* transformation. Four proposed transformations (*r*, *rv*, *r2*, and *rv2*) are based on root transformations. The other 4 proposed transformations (*l*, *lv*, *l2*, and *lv2*) are based on log transformations. The transformations *r*, *rv*, *l*, and *lv* do the same transformation to each read count, while the transformations *r2*, *rv2*, *l2*, and *lv2* are sample-specific (i.e., each sample has its own transformation, like *voom*). To evaluate the effects of sample size on the performances of limma with data transformed by each of the 8 proposed data transformations and to compare them with the performance of limma with data transformed by the *voom* transformation, we performed eight simulation studies based on the simulation scheme in^12^. Since real datasets seldom have equal library size, we only consider to simulate datasets with un-equal library sizes in our simulation studies.

In addition, we would like to evaluate if using non-parametric approaches would have better performance than using parametric approaches in analyzing RNA-seq data, the distribution of which is non-normal. Specifically, we applied the *Wilcoxon rank sum* test (denoted it as *Wilcoxon*) for each gene transcript based on the *untransformed* counts. We then adjusted p-values to control false discovery rate < 0.05.

**Figure 2:**
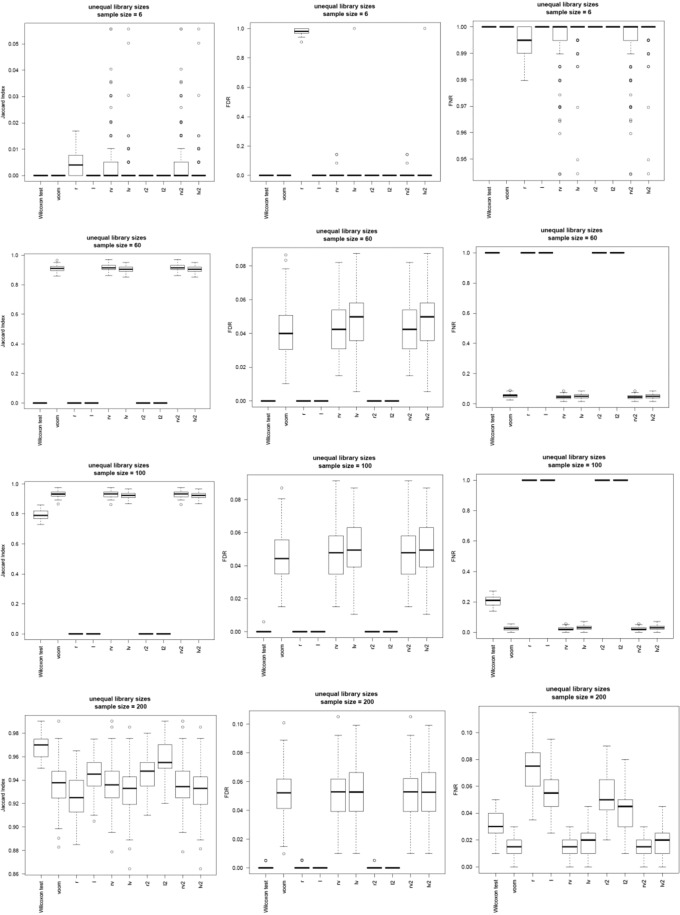
Results based on the 100 simulated datasets with unequal library size. Upper horizontal panel: nCases = nControls = 3; Second horizonal panel: nCases = nControls = 30; Third horizontal panel: nCases = nControls = 50; Bottom horizontal panel: nCases = nControls = 100. Left column: Jaccard index; Middle column: FDR; Right column: FNR.

Figure 2 and Supplementary Fig. 1 show that for data sets with small (e.g., nCases = nControls = 3) or large sample size (e.g., nCases = nControls = 100) limma based on datasets from the *l*, *l2*, and *r2* transformations performed better than limma based on datasets from the *voom* transformation in term of accuracy, false discovery rate (FDR), and false discovery rate (FNR). For datasets with moderate sample sizes (e.g., nCases = nControls = 30 or 50), limma with the *rv* and *rv2* transformations performed similarly to limma with the *voom* transformation. The accuracy is measured by the Jaccard index, which is defined as the ratio *d*/(*b* + *c* + *d*), where *d* is the number of truly differentially expressed (DE) gene transcripts having been detected as DE gene transcripts, *b* is the number of truly DE gene transcripts having been detected as non-DE gene transcripts, *c* is the number of truly non-DE gene transcripts having been detected as DE gene transcripts. The Jaccard index is a better measurement than commonly-used accuracy measurement *acc* = (*a* + *d*)/(*a* + *b* + *c* + *d*), where a is the number of truly non-DE gene transcripts having detected as non-DE gene transcripts, for datasets with highly imbalanced proportions (i.e., *a* is much larger than *d*) of truly DE gene transcripts and truly non-DE gene transcripts. When *a* is much larger than *d*, then *a* will dominate in the calculation of *acc*. Hence, *acc* tends to close to one, which is misleading. FDR is the proportion of truly non-DE gene transcripts among detected DE gene transcripts. FNR is the proportion of detected non-DE gene transcripts among truly DE gene transcripts. A gene transcript is detected as a DE gene transcript if its FDR adjusted p-value is <0.05; it is detected as a non-DE gene transcript if its FDR adjusted p-value ≥0.05.

Surprisingly, the *Wilcoxon* test without data transformation performed best in terms of accuracy when the sample size was large and samples had unequal library sizes in our simulation studies, in which we used Law *et al*.*’s*^12^ simulation setting. *Wilcoxon* also had the lowest FDR and low FNR. However, *Wilcoxon* had significant higher FNR than limma with *voom*, *rv*, *lv*, *rv2*, or *lv2*, indicating that *Wilcoxon* had lower power than limma with *voom*, *rv*, *lv*, *rv2*, or *lv2*.

Overall, the *Wilcoxon* test without data transformation can be used to detect differentially expressed gene transcripts for RNA-seq data when sample size is large and sample library sizes are unequal. If data transformation is preferred, then limma with *voom*, *l, r2*, or *lv2* can be used in this scenario.

Supplementary Fig. 2 showed the boxplots of the estimated model parameters for the 100 simulated datasets for each of the 4 sample-size scenarios. For small-sample-size scenarios (nCases = nControls = 3), the estimated parameters are much larger and variable than scenarios with larger sample sizes, in which the median parameter estimates are almost unchanged as sample size increases. Also, the variabilities of the parameter estimates are similar for scenarios with sample size ≥ 60. For the 4 proposed root transformations, the medians of the estimated *η* are similar and are ranged from around 5.7 to 9.5. For the 4 proposed log transformations, the medians of the estimated *δ* are similar and are ranged from around 0.08 to 0.32.

**Figure 3:**
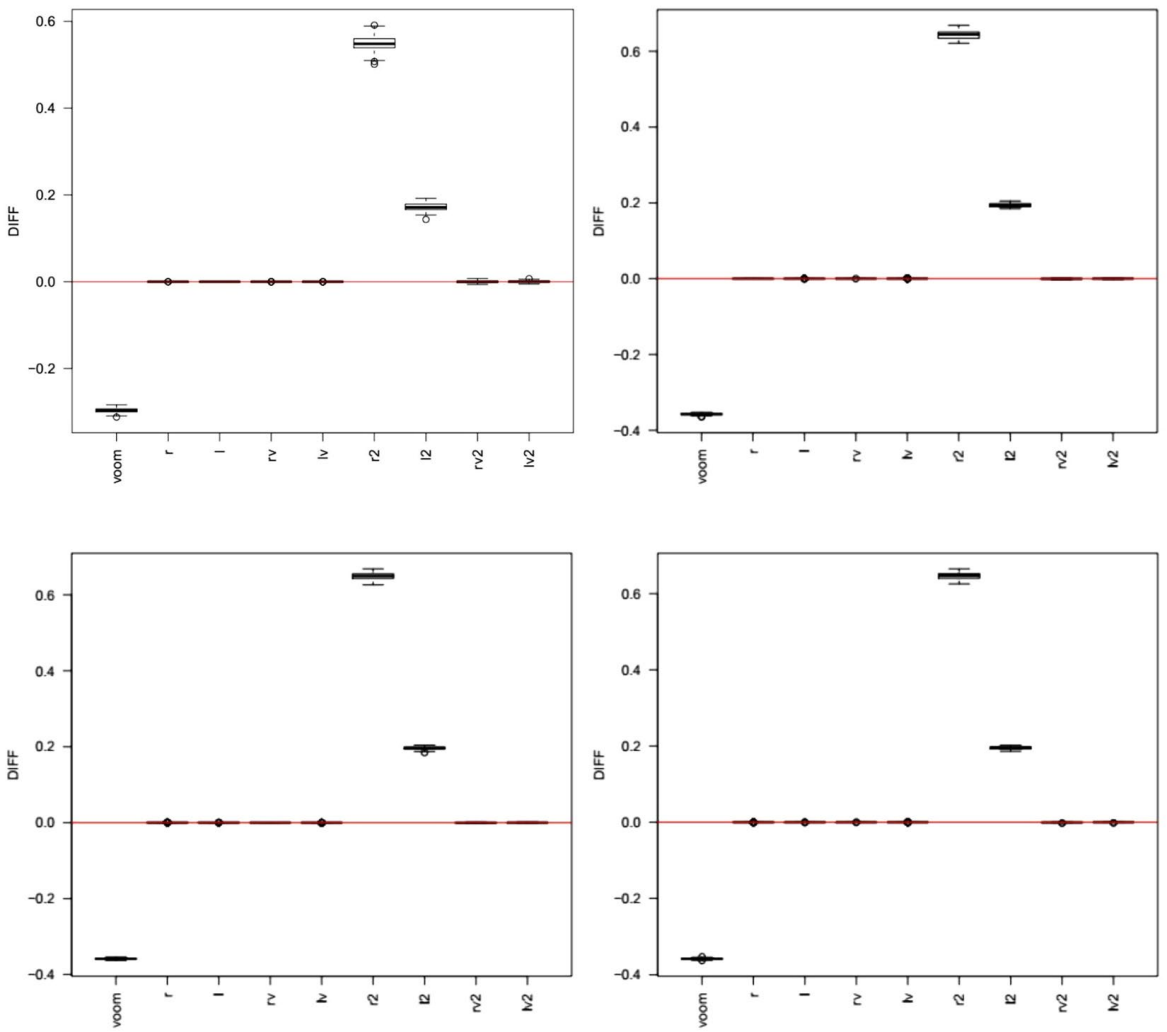
The difference (y-axis) between sample mean and sample median of the pooled expression levels of all gene transcripts and all samples after data transformation in our simulation studies. Top left panel: nCases = nControls = 3. Top right panel: nCases = nControls = 30; Bottom left panel: nCases = nControls = 50; Bottom right panel: nCases = nControls = 100.

As we mentioned in the Background section, we aimed to transform the RNA-seq count data so that the sample mean would be representative of the center of the transformed empirical distribution. Hence, the difference between sample mean and sample median after transformation is an important judging criterion of RNA-seq transformation methods. We used the difference between sample mean and sample median based on the pooled expression levels of all gene transcripts and all samples to check if sample mean is close to the sample median (Fig. 3). The smaller the difference, the closer the sample mean is to the distribution center. Figure 3 showed that *r*, *l*, *rv*, *lv*, *rv2*, and *lv2* had the mean-median difference close to zero. However, *r2* and *l2* had much larger mean-median difference than zero. This is as what we expected since *r2* and *l2* aims to minimize the sum of sample-specific squared difference between sample mean and sample median, not to minimize the squared difference between sample mean and sample median of the pooled data. While *l2* had smaller difference than *voom*, the *r2* transformation had much larger mean-median difference than *voom*. Supplementary Fig. 3 showed the boxplots of the sum of sample-wise squared differences between sample mean and sample median after data transformation in our simulation studies. *r2* and *l2* transformations had much smaller mean-median difference than *r* and *l* transformations.

### Real data analysis

**Figure 4:**
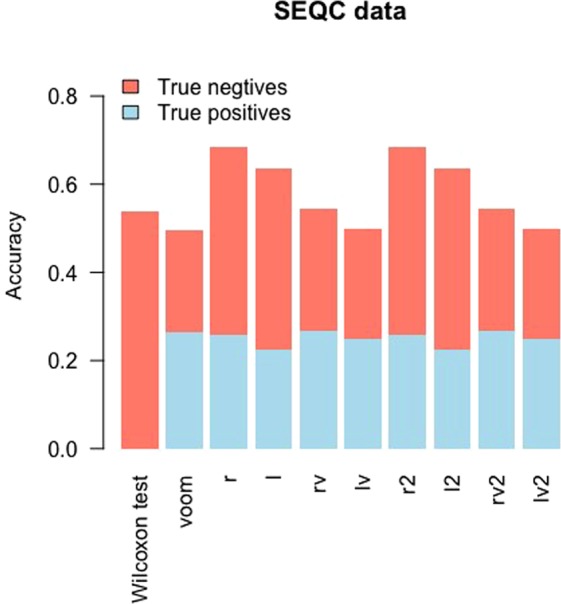
Accuracy for the SEQC dataset. The accuracy (acc) is plotted for each method and is split into true positive rate and true negative rate. Limma with the *r* and *r2* transformations had the highest accuracy (0.6836).

The first two of our real data analysis datasets were based on the SEQC datasets^17^. We applied the *Wilcoxon* test, *limma* after the TMM scale normalization^18^, quantile normalization, and the *voom* transformation (We still denoted the method as limma with *voom*), and *limma* after the 8 proposed transformations to the SEQC RNA-seq dataset. The information about which gene transcripts are truly differentially expressed between two groups were determined based on qRT-PCR (Quantitative Real-Time PCR) experimental data. Figure 4 showed that limma with *r*, *l*, *rv, r2*, *l2, rv2* had higher accuracy than limma with *voom* and showed that limma with *lv* and *lv2* had equal accuracies to limma with *voom*. We noticed that *Wilcoxon*, a non-parametric method, failed to detect any true positives, although *Wilcoxon* had slighly higher accuracy to limma with *voom*. Since we know the true DE status of each gene transcript in SEQC dataset, we calculated the FDR and FNR values for the 10 methods (Supplementary Table 1). For SEQC dataset (nCases = nControls = 4), *rv* and *rv2* transformations had lower FDR and FNR than *voom*.

**Figure 5:**
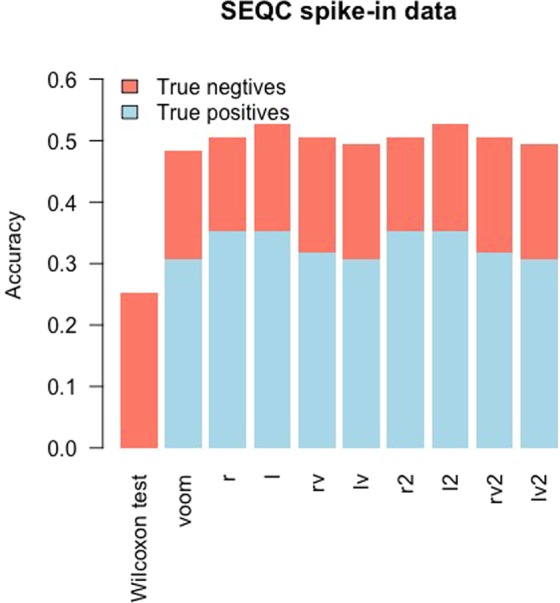
Accuracies obtained based on the SEQC spike-in data. The accuracy (=true positives + true negatives) is plotted for each of the 10 methods. Limma with the *l2* and *l* transformations had the highest accuracies (0.53).

The analysis of the ERCC dataset is consistent with the SEQC analysis. The information about which gene transcripts are truly differentially expressed between two groups were determined based on concentrations of mixes (see Method Section). Figure 5 showed that limma with the *r*, *l*, *rv*, *r2*, *l2*, and *rv2* transformations had better accuracies than limma with *voom* and showed that limma with the *lv* and *lv2* transformations had equal accuracies to limma with *voom*. Specifically, limma with *r*, *l*, *rv*, *r2*, *l2*, and *rv2* had more true positives than limma with *voom*, indicating good testing powers. As in the analysis in the SEQC data, the *Wilcoxon* test failed to detect any true positives and had the lowest accuracy, which is consistent with the results of the simulation studies, indicating the *Wilcoxon* test has poor performance in datasets with small sample sizes. Since we know the true DE status of each gene transcript in ERCC dataset, we calculated the FDR and FNR values for the 10 methods (Supplementary Table 2). For ERCC dataset (nCases = nControls = 4), *rv* and *rv2* transformations had lower FDR and FNR than *voom*.

**Figure 6:**
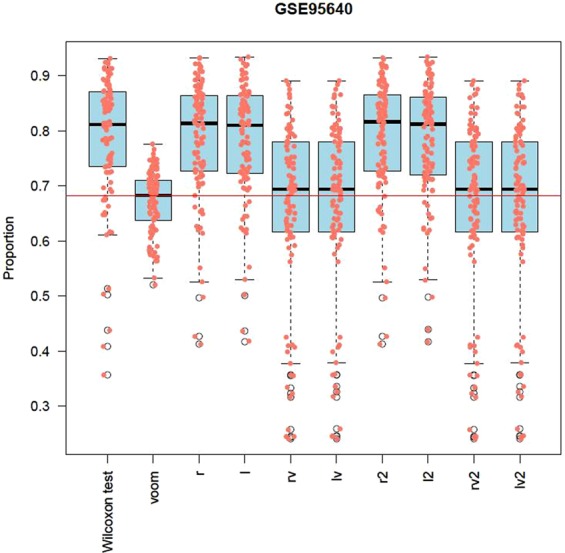
Parallel boxplots of the proportion of validated gene transcripts in the analysis of GSE95640. We did 100 randomly splits the 382 samples. In each split, we randomly split the 382 samples into roughly equal two parts: discovery set and validation set. The proportion of the significant DE gene transcripts detected in the discovery set and validated in the validation set was recorded for each split. Each boxplot is the summary of the proportions of validated gene transcripts for the 100 pairs of discovery sets and validation sets. The higher the proportion, the better the performance.

GSE95640 is an RNA-seq dataset from a study investigating adipose tissue during low-caloric diet (LCD) that has relatively large sample size (n = 382) with 53343 gene transcripts. We used all 382 samples (191 samples from after 8-week LCD (with 800–1000 kcal/d) and 191 samples from 6-month after LCD) and conducted 100 random partitions of the 382 samples. In each random partition, we randomly split the 382 samples into roughly 2 equal sets: discovery set and validation set. We then calculated the proportion of validated DE gene transcripts for each of the 10 methods. Figure 6 showed that limma with *r, l, r2*, and *l2* had higher median validation proportion than *Wilcoxon* and limma with other transformations in two analyses. Note that GSE95640 dataset is from a paired design. There are 191 subjects. Each subject has two observations (after 8 weeks and after 6 months). Within a subject, the two observations are dependent. However, GSE95640 dataset does not provide subject id info. Hence, we ignored the within-subject correlations in this real data analysis. As a consequence, less numbers of DE gene transcripts would be detected than the analyses in which subject id info is known and statistical tests for paired data are applied.

**Figure 7:**
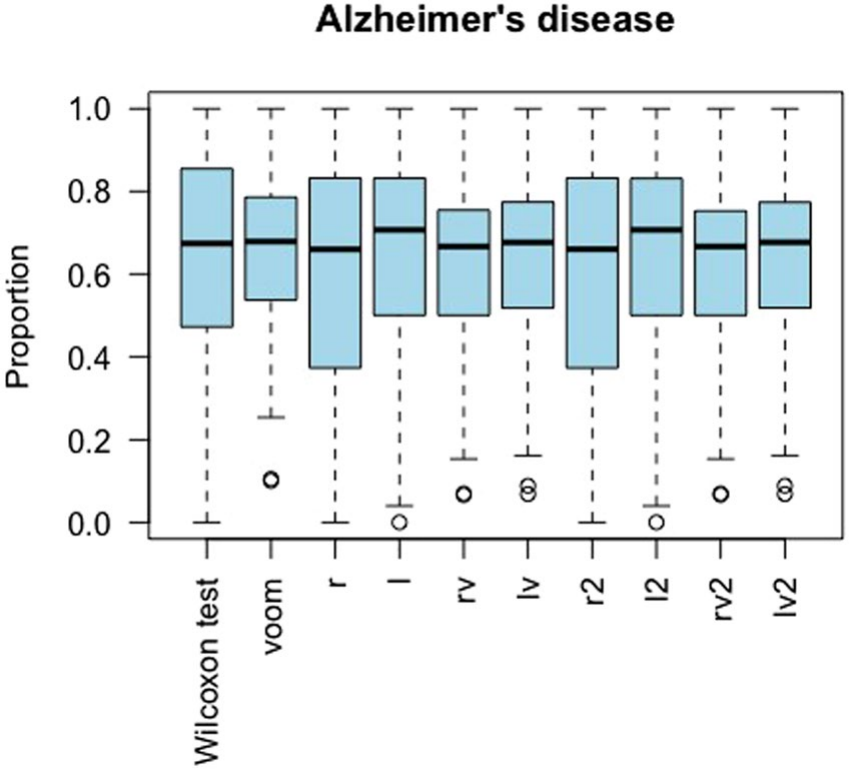
Parallel boxplots of the proportion of validated genes in the analysis of GSE95587. We did 100 randomly splits the 117 samples. In each split, we randomly split the 117 samples into roughly equal two parts: discovery set and validation set. The proportion of the significant DE gene transcripts detected in the discovery set and validated in the validation set was recorded for each split. Each boxplot is the summary of the proportions of the 100 proportions. The higher the proportion, the better the performance.

GSE95587 is an RNA-seq dataset from a study investigating Alzheimer’s disease that has relatively large sample size (n = 117). We used all 117 samples (84 Alzheimer samples and 33 age-matched normal controls) and conducted 100 random partitions of the 117 samples. In each random partition, we randomly split the 117 samples into roughly 2 equal sets: discovery set and validation set. We then calculated the proportion of validated DE gene transcripts for each of the 10 methods. Figure 7 showed that all 10 methods had similar validation proportion, with *l* and *l2* had slightly higher median proportion of validation than *voom*.

## Discussion

In this article, we proposed 8 new RNA-seq data transformations to improve the *voom* transformation for RNA-seq data analysis. The simulation results showed that for data sets with small (e.g., nCases = nControls = 3) or large sample size (e.g., nCases = nControls = 100) limma based on data from the *l*, *l2*, and *r2* transformations performed better than limma based on data from the *voom* transformation in term of accuracy, FDR, and FNR. For datasets with moderate sample size (e.g., nCases = nControls = 30 or 50), limma with the *rv* and *rv2* transformations performed similarly to limma with the *voom* transformation.

Having sample mean close to sample median for pooled data could not guarantee that for each gene transcript, the sample mean is close to the sample median for cases and for controls, respectively. Also, the empirical distribution of the pooled data after data transformation might still be skewed even if the sample mean is very close to sample median. Hence, robust linear regression might improve the performance of limma after data transformation.

The *voom* transformation was proposed by^12^ and has been implemented in the *limma* package. Law *et al*.^12^ focused on small sample size (nCases = nControls = 3) because RNA-seq data were expensive to obtain at that time. Since then the cost of RNA sequencing become lower and lower. Hence, we evaluated the performances of limma with *voom* and limma with the 8 new transformations in scenarios where sample sizes are relatively large (nCases = nControls = 100). We also applied the *Wilcoxon* test to the *raw count* data to check if data transformation could perform better than the non-parametric test. Interestingly, the *Wilcoxon* test *without data transformation* performed better than the *limma* analysis based on the 9 data transformations in simulation studies when sample sizes are not too small and sample library sizes are unequal. The analysis of the GSE95640 dataset also demonstrated this finding. Further investigation is warranted.

In this article, we did not compare *limma* with count-based methods, such as edgeR and DESeq since Law *et al*.^12^ did the comparison and showed the good performance of the data transformation approach. However, Law *et al*.^12^ did the comparisons based on datasets with small sample sizes. Moreover, new count-based methods, such as DESeq2, have been proposed since 2014. Hence, it would be a future research to compare the data transformation approach with all available count-based methods using datasets with large sample sizes (e.g., nCases = nControls = 1000).

We also did not compare the two useful RNAseq analysis tools: NOIseq^19^ and sleuth^20^ since this article focuses on comparing count transformation methods in RNAseq analysis, while NOIseq and sleuth provide methods for differential expression analysis. NOIseq R package provides useful tools for quantifying gene expression, assessing the quality of the expression data, choosing appropriate normalization or filtering methods according to the biases detected, performing non-parametric differential expression analysis, and visualizing the results. Sleuth utilizes kallisto quantification^21^ and bootstrapping and response error linear modeling to detect differentially expressed genes. It would be an interesting future research topic to compare NOIseq, Sleuth, and limma combined with different count transformation methods.

We observed that none of the 8 transformations could dominate each other, although they performed better than *voom* in most scenarios (Fig. 2 and Supplementary Table 1). For example, limma with the *r2* transformation performed best when sample size is large (nCases = nControls = 100), but could not beat limma with *rv* when sample size is moderate (e.g., nCases = nControls = 30 or 50). Another limitation of our study is that in our real data analyses, no independent cohorts are available to do validation. However, we did 100 times of random splits. In each split, we had discovery set and validation set. In future, we will do validation studies when independent validation sets are available. The third limitation of this study is that the 8 proposed data transformations aim to make the sample mean closer to the distribution center after data transformation. However, the transformed distribution might not be close to a normal distribution. Hence, robust linear regression models are needed since ordinary linear regression requires the normality assumption. Future research is warranted on this subject as well.

In the simulation studies, we considered the scenario with small sample size (nCases = nControls = 3), which could not have adequate power. The main reasons why we consider this scenario are (1) Law *et al*. (2014) investigated this scenario; and (2) pilot studies usually have small sample sizes. We also considered the scenario with large sample size (nCases = nControls = 100), which are rare in real applications due to the high expenses to obtain RNA-seq data. The main reasons why we consider this scenario are (1) it would be interesting to know the performances of different methods for datasets with large sample size; and (2) in some real application, sample sizes are large, e.g., GSE95640 (nCases = nControls = 191).

We observed from both simulation and real data analyses that when sample size is moderate (e.g., the Alzheimer’s disease dataset GSE95587), all the 8 proposed data transformation methods could not beat voom transformation. Further investigation is warranted.

## Conclusions

In simulation and real data studies, limma with the *l*, *l2*, and *r2* transformations performed better than limma with the *voom* transformation for data with small (nCases = nControls = 3) or large sample size (nCases = nControls = 100). For moderate sample size (nCases = nControls = 30 or 50), limma with the *rv* and *rv2* transformation performed better than limma with the *voom* transformation. We hope these novel data transformations could provide investigators more powerful differentially expression analysis using RNA-seq data.

## Materials and Methods

### Eight new data transformations

We proposed 8 new data transformations based on the Box-Cox transformation^22^: 4 root transformations (denoted as *r*, *rv*, *r2* and *rv2*, respectively) and 4 log transformations (denoted as *l*, *lv*, *l2* and *lv2*, respectively). The following two properties of the root transformation motivate us to use root transformations: (1) root transformation of zero exists; (2) root transformation could stabilize the variance of count data.

The *r* transformation (root transformation) is defined as:

$${y}_{gi}=\frac{{{x}_{gi}}^{(1/\eta )}}{(1/\eta )}$$

where *x*_*gi*_ is the count of the *g*-th gene transcript for the *i*-th sample. The optimal value for the parameter *η* is to minimize the difference between the sample mean and the sample median of the pooled data:

$${\eta }_{opt}={argmi}{{n}}_{\eta }{[\bar{y}-\tilde{y}]}^{2},$$

where $\bar{y}=\sum _{i=1}^{n}\,\sum _{g=1}^{G}\,{y}_{gi}/(nG)$ is the sample mean and $\tilde{y}$ is the sample median of the pool data *y*_*gi*_, *i* = 1, …, *n*, *g* = 1, …, *G*. That is, for a given *η*, we perform the *r* transformation to each count *x*_*gi*_, *i* = 1, …, *n*, *g* = 1, …, *G*. We then can obtain the squared difference ${(\bar{y}-\tilde{y})}^{2}$. Finally, we choose the value of *η* having minimum squared difference.

The *rv* transformation (root and voom transformation) is defined as:

$${y}_{gi}=\frac{{t}_{gi}^{(1/\eta )}}{(1/\eta )}$$

where $${t}_{gi}=\frac{({x}_{gi}+0.5)}{{X}_{i}+1}\times {10}^{6}$$, $${X}_{i}=\sum _{g=1}^{G}\,{x}_{gi}$$.

That is, we do the root transformation for the sample-specific counts per million. The optimal value for the parameter *η* is to minimize the squared difference between the sample mean and the sample median of the pooled data. That is, for a given *η*, we perform the *rv* transformation to each normalized count *t*_*gi*_, *i* = 1, …, *n*, *g* = 1, …, *G*. We then can obtain the squared difference ${(\bar{y}-\tilde{y})}^{2}$, where $\bar{y}=\sum _{i=1}^{n}\,\sum _{g=1}^{G}\,{y}_{gi}/(nG)$ is the sample mean and $\tilde{y}$ is the sample median of the pool data *y*_*gi*_, *i* = 1, …, *n*, *g* = 1, …, *G*. Finally, we choose the value of *η* having minimum squared difference.

The *r2* transformation (root transformation minimizing sum of sample-specific squared difference) has the same form as the *r* transformation:

$${y}_{gi}=\frac{{{x}_{gi}}^{(1/\eta )}}{(1/\eta )}$$

However, the criterion to estimate the optimal value of *η* is different from the *r* and *rv* transformations. The optimal value for the parameter *η* is to minimize the sum of the squared difference between the sample mean and the sample median across *n* samples:

$${\eta }_{opt}={argmi}{{n}}_{\eta }\sum _{i=1}^{n}\,{[{\bar{y}}_{i}-{\tilde{y}}_{i}]}^{2}$$

where ${\bar{y}}_{i}=\sum _{g=1}^{G}\,{y}_{gi}/G$ is the sample mean expression level for the *i*-th sample and ${\tilde{y}}_{i}\,$ is the sample median expression level of the *i*-th sample.

The *rv2* transformation (root and voom transformation minimizing sum of sample-specific squared difference) is a combination of the *rv* transformation and the *r2* transformation, defined as:

$${y}_{gi}=\frac{{t}_{gi}^{(1/\eta )}}{(1/\eta )}$$

where *t*_*gi*_ is the sample-specific counts per million. The optimal value for the parameter *η* is to minimize the sum of the squared difference between the sample mean and the sample median across *n* samples.

The *l* transformation (log transformation) is defined as:

$${y}_{gi}={\mathrm{log}}_{2}({x}_{gi}+\frac{1}{\delta })$$

The optimal value for the parameter *δ* is to minimize the squared difference between the sample mean and the sample median of the pooled data:

$${\delta }_{opt}={argmi}{{n}}_{\delta }{[\bar{y}-\tilde{y}]}^{2},$$

where $\bar{y}=\sum _{i=1}^{n}\,\sum _{g=1}^{G}\,{y}_{gi}/(nG)$ is the sample mean and $\tilde{y}$ is the sample median of the pool data *y*_*gi*_, *i* = 1, …, *n*, *g* = 1, …, *G*.

The *lv* transformation (log and voom transformation) is defined as:

$${y}_{gi}={\mathrm{log}}_{2}({t}_{gi}+\frac{1}{\delta })$$

where *t*_*gi*_ is the sample-specific counts per million. The optimal value for the parameter *δ* is to minimize the squared difference between the sample mean and the sample median of the pooled data.

The *l2* transformation (log transformation minimizing sum of sample-specific squared difference) has the same form as the *l* transformation:

$${y}_{gi}={\mathrm{log}}_{2}({x}_{gi}+\frac{1}{\delta })$$

However, the criterion to estimate the optimal value of *δ* is different from the *l* and *lv* transformations. The optimal value for the parameter *δ* is to minimize the sum of the squared difference between the sample mean and the sample median across *n* samples:

$${\delta }_{opt}={argmi}{{n}}_{\delta }\sum _{i=1}^{n}\,{[{\bar{y}}_{i}-{\tilde{y}}_{i}]}^{2},$$

where ${\bar{y}}_{i}=\sum _{g=1}^{G}{y}_{gi}/G$ is the sample mean expression level for the *i*-th sample and ${\tilde{y}}_{i}\,$ is the sample median expression level of the *i*-th sample.

The *lv2* transformation (log and voom transformation minimizing sum of sample-specific squared difference) is a combination of the *lv* transformation and the *l2* transformation, defined as:

$${y}_{gi}={\mathrm{log}}_{2}({t}_{gi}+\frac{1}{\delta })$$

where *t*_*gi*_ is the sample-specific counts per million. The optimal value for the parameter *δ* is to minimize the sum of the squared difference between the sample mean and the sample median across *n* samples.

### Simulation studies

In each simulation study, we generated 100 datasets. Each dataset contains 10,000 genes, among which 200 genes are differentially expressed between *nCases* cases and *nControls* controls. An inverse chi-square distribution with 40 degrees of freedom was used to generate a modest amount of gene-wise biological variation^12^. We set the number of cases equal to the number of controls (i.e., nCases = nControls).

After data transformation, we used Bioconductor package *limma* to detect differentially expressed genes. We also compared the results based on transformed data with the results of the *Wilcoxon* rank sum test *based on the original counts*.

Since almost all real RNAseq datasets have unequal library sizes, we applied Law *et al*.*’s*^12^ simulation settings to generate the RNA-seq counts of 10,000 genes for samples with un-equal library sizes. In our simulation studies, we evaluated the effects of sample size on the performances of the 10 methods (*Wilcoxon*, *limma + voom*, *limma + r*, *limma + l*, *limma + r2*, *limma + l2*, *limma+rv*, *limma+lv*, *limma+rv2*, *limma+lv2*) in detecting differentially expressed genes. We tried four different sample sizes (nCases = nControls = 3, nCases = nControls = 30, nCases = nControls = 50 and nCases = nControls = 100) with un-equal library size, respectively.

The criteria to evaluate performance are accuracy (Jaccard index), false negative rate (FNR), false discovery rate (FDR), and the difference (DIFF) between the sample mean and the sample median of the pooled expression levels for all samples and all genes. The accuracy is measured by the Jaccard index, which is defined as the ratio *d*/(*b* + *c* + *d*), where *d* is the number of truly differentially expressed (DE) genes having been detected as DE genes, *b* is the number of truly DE genes having been detected as non-DE genes, *c* is the number of truly non-DE genes having been detected as DE genes. FNR is the percentage of detected non-differentially expressed (non-DE) genes among truly differentially expressed (DE) genes. FDR is the percentage of truly non-DE genes among detected DE genes. Large accuracy and small FNR, FDR, and DIFF indicate good performance.

### Real data analyses

#### SEQC data

Sequencing Quality Control (SEQC) is the third phase of the MAQC project (MAQC-III), aimed at assessing the technical performance of next-generation sequencing platforms by generating benchmark datasets with reference samples^17,23^. This project provided 6 RNA samples, each sample has 4 replicates, samples A and B were obtained from two well-characterized reference human RNA samples UHR (Universal Human Reference RNA) and HBR (Human Brain Reference RNA). A small amount of Ambion ERCC (External RNA Control Consortium) Spike-in Mix was added into both Sample A and Sample B. Samples C and D were constructed by mixing Samples A and B to known ratios, 3:1 and 1:3, respectively. The pure ERCC Spike-in Mix 1 and 2 were used as Samples E and F. Gene expression levels of Samples A, B, C and D were analyzed by using TaqMan RT-PCR technology.

Our first analysis is based on Samples A (UHR) and B (HBR). The dataset GSE56457 on Gene Expression Omnibus (GEO) website (https://www.ncbi.nlm.nih.gov/geo/query/acc.cgi?acc=GSE56457) provides details about the qRT-PCR data for the SEQC project. We regarded the expression levels of these genes measured by qRT-PCR as the true expression levels. If a gene has mean log2 fold-change (LFC) greater than 2 between two RNA samples in GSE56457, we claimed it as a truly differentially expressed gene. If a gene has mean LFC less than 0.004, we claimed it as a truly non-differentially expressed gene^24^. Based on this criterion, there were 390 DE genes and 457 non-DE genes. We evaluated the performance of the 9 data transformations using SEQC data based on these 847 genes. We applied *limma* to detect differentially expressed genes after data transformation. We also applied the *Wilcoxon* test to detect DE genes based on the raw count data. A gene was estimated as a DE gene if it had FDR-adjusted p-value < 0.05. We then calculated the proportion of agreement (i.e., accuracy) between the true gene significance of the 847 genes and the estimated gene significance.

### SEQC spike-in (ERCC)

We downloaded ERCC data from http://bioinf.wehi.edu.au/voom/ and did similar analysis based on Samples E and F, which are the ERCC RNA Spike-In Mixes, providing a set of external RNA controls that enable performance assessment of a variety of technology platforms used for gene expression experiments. These 8 samples (4 from Samples E and 4 from Samples F) are pre-formulated sets of 92 poly adenylated genes from the ERCC plasmid reference library, three quarters of the genes were truly DE and the remaining quarter were not. The genes are traceable through the manufacturing process to the NIST plasmid reference material. This dataset provided concentrations of the two mixes, the log2 fold change of concentration can be used for determining if a gene is DE. The analysis procedure of spike-in data is consistent with SEQC data. We calculated the accuracy to compare the transformation methods performance.

### Low-caloric diet (LCD) RNA samples

GSE95640 (https://www.ncbi.nlm.nih.gov/geo/query/acc.cgi?acc=GSE95640) is an RNA-seq dataset to evaluate transcriptome alterations in adipose tissue (AT) during LCD based on 191 obese, non-diabetic patients. This RNA-seq dataset is sequenced on Illumina HiSeq 2000 platform. We downloaded the RNA-seq raw data and annotations from Gene Expression Omnibus (GEO, https://www.ncbi.nlm.nih.gov/geo). The dataset contains 382 samples with 53343 gene transcripts, 191 of which are from the transcriptome after 8-week LCD (with 800–1000 kcal/d) (CID1) and 191 of which are from the transcriptome 6-month after LCD (CID2). We randomly split the 392 samples into two roughly equal parts: a discovery set and a validation set. We then applied *limma* after data transformations to the discovery set and the validation set to detect differentially expressed (DE) genes. For the discovery set, we claimed a gene is DE if its FDR-adjusted p-value < 0.05. For the validation set, we claimed a gene is validated DE if it had a raw p-value < 0.05 in the validation set and it had FDR-adjusted p-value < 0.05 in the discovery set. We repeated the above split-validation procedure 100 times. For each of the 10 methods, we calculated the proportion of the validated DE genes among the DE genes detected in the discovery set. The higher the proportion is, the better performance, the method is.

### Neurodegenerative disease RNA samples

GSE95587 (https://www.ncbi.nlm.nih.gov/geo/query/acc.cgi?acc=GSE95587) is an RNA-seq dataset obtained from fusiform gyrus tissue sections of autopsy-confirmed Alzheimer’s cases and neurologically age-matched normal controls. The matching information was not provided in GSE95587. We downloaded the RNA-seq raw data and annotations from Gene Expression Omnibus (GEO, https://www.ncbi.nlm.nih.gov/geo). The dataset contains 117 samples, 84 of which are from Alzheimer’s cases (ADs) and 33 of which are controls (CONs). We randomly split the 117 samples into two roughly equal parts: a discovery set and a validation set. The discovery set has 42 ADs and 17 CONs. The validation set has 42 ADs and 16 CONs. We then applied *limma* after data transformations to the discovery set and the validation set to detect differentially expressed (DE) genes. For the discovery set, we claimed a gene is DE if its FDR-adjusted p-value < 0.05. For the validation set, we claimed a gene is validated DE if it had a raw p-value < 0.05 in the validation set and it had FDR-adjusted p-value < 0.05 in the discovery set. We repeated the above split-validation procedure 100 times. For each of the 10 methods, we calculated the proportion of the validated DE genes among the DE genes detected in the discovery set. The higher the proportion is, the better performance, the method is.

For the *voom* transformation in all real data analyses in this article, we followed Law *et al*.^12^ by first applying TMM scale-normalization^18^ and quantile normalization before applying for the *voom* transformation.

## Supplementary information

Supplementary Document

## Data Availability

The SEQC data can be downloaded from Gene Expression Omnibus (GEO) with accession number GSE56457 (https://www.ncbi.nlm.nih.gov/geo/query/acc.cgi?acc=GSE56457). The SEQC spike-in (ERCC) data can be downloaded from http://bioinf.wehi.edu.au/voom/. The LCD data can be downloaded from GEO with accession number GSE95640 (https://www.ncbi.nlm.nih.gov/geo/query/acc.cgi?acc=GSE95640). The neurodegenerative disease data can be downloaded from GEO with accession number GSE95587 (https://www.ncbi.nlm.nih.gov/geo/query/acc.cgi?acc=GSE95587). We developed the R package *countTransformers*, which can be downloaded from CRAN website https://CRAN.R-project.org/package=countTransformers.

## References

[1] Mortazavi A, Williams BA, McCue K, Schaeffer L, Wold B (2008). Mapping and quantifying mammalian transcriptomes by RNA-Seq. Nat Methods..

[2] Wang Z, Gerstein M, Snyder M (2009). RNA-Seq: a revolutionary tool for transcriptomics. Nat Rev Genet..

[3] Marioni JC, Mason CE, Mane SM, Stephens M, Gilad Y (2008). RNA-seq: an assessment of technical reproducibility and comparison with gene expression arrays. Genome Res..

[4] Marguerat S, Bähler J (2010). RNA-seq: from technology to biology. Cell Mol Life Sci..

[5] Cloonan N (2008). Stem cell transcriptome profiling via massive-scale mRNA sequencing. Nat Methods..

[6] Auer, P. & Doerge, R. A two-stage Poisson model for testing RNA-seq data. *Statistical Applications in Genetics and Molecular Biology*. **10**, Article 26 (2011).

[7] Li J, Witten DM, Johnstone IM, Tibshirani R (2012). Normalization, testing, and false discovery rate estimation for RNA-sequencing data. Biostatistics..

[8] Robinson MD, Smyth GK (2007). Moderated statistical tests for assessing differences in tag abundance. Bioinformatics..

[9] McCarthy DJ, Chen Y, Smyth GK (2012). Differential expression analysis of multifactor RNA-Seq experiments with respect to biological variation. Nucleic Acids Res..

[10] Anders S, Huber W (2010). Differential expression analysis for sequence count data. Genome Biol..

[11] Love MI, Huber W, Anders S (2014). Moderated estimation of fold change and dispersion for RNA-seq data with DESeq2. Genome Biol..

[12] Law CW, Chen Y, Shi W, Smyth GK (2014). voom: Precision weights unlock linear model analysis tools for RNA-seq read counts. Genome Biol..

[13] Ritchie ME (2015). limma powers differential expression analyses for RNA-sequencing and microarray studies. Nucleic Acids Res..

[14] Hardcastle TJ, Kelly KA (2010). baySeq: empirical Bayesian methods for identifying differential expression in sequence count data. BMC Bioinformatics..

[15] Wu H, Wang C, Wu Z (2013). A new shrinkage estimator for dispersion improves differential expression detection in RNA-seq data. Biostatistics..

[16] Phipson B, Lee S, Majewski IJ, Alexander WS, Smyth GK (2016). Robust hyperparameter estimation protects against hypervariable genes and improves power to detect differential expression. Ann Appl Stat..

[17] Su Z (2014). A comprehensive assessment of RNA-seq accuracy, reproducibility and information content by the Sequencing Quality Control Consortium. Nat Biotechnol..

[18] Robinson MD, Oshlack A (2010). A scaling normalization method for differential expression analysis of RNA-seq data. Genome Biol..

[19] Tarazona S (2015). Data quality aware analysis of differential expression in RNA-seq with NOISeq R/Bioc package. Nucleic Acids Res..

[20] Pimentel H, Bray NL, Puente S, Melsted P, Pachter L (2017). Differential analysis of RNA-seq incorporating quantification uncertainty. Nat Methods..

[21] Bray NL, Pimentel H, Melsted P, Pachter L (2016). Near-optimal probabilistic RNA-seq quantification. Nat Biotechnol..

[22] Box G, Cox D (1964). An analysis of transformations. Journal of the Royal Statistical Society Series B (Methodological)..

[23] Sequencing Quality Control (SEQC) Project. https://www.fda.gov/ScienceResearch/BioinformaticsTools/MicroarrayQualityControlProject/default.htm#MAQC-IIIalsoknownasSEQC (2014).

[24] Canales RD (2006). Evaluation of DNA microarray results with quantitative gene expression platforms. Nat Biotechnol..

