# Supplementary material for: Novel Data Transformations for RNA-seq Differential Expression Analysis
Source: Sci Rep. 2019 Mar 18;9:4820. doi: 10.1038/s41598-019-41315-w (PMC6423143; doi:10.1038/s41598-019-41315-w)
Supplement: Supplementary file 1 — Supplementary Document [file 41598_2019_41315_MOESM1_ESM.pdf]

Supplementary Document

Novel Data Transformations for RNA-seq Differential Expression Analysis

Zeyu Zhang[1], Danyang Yu[2], Minseok Seo[3], Craig P. Hersh[3], Scott T. Weiss[3], Weiliang Qiu*[3]

[1] Department of Bioinformatics, School of Life Sciences and Technology, Tongji University, Shanghai, China
[2] Department of Information and Computing Science, College of Mathematics and Econometrics, Hunan University, Hunan, China
[3] Channing Division of Network Medicine, Brigham and Women's Hospital/ Harvard Medical School, Boston, USA
[*] Corresponding author’s email address: stwxq@channing.harvard.edu

Supplementary Document

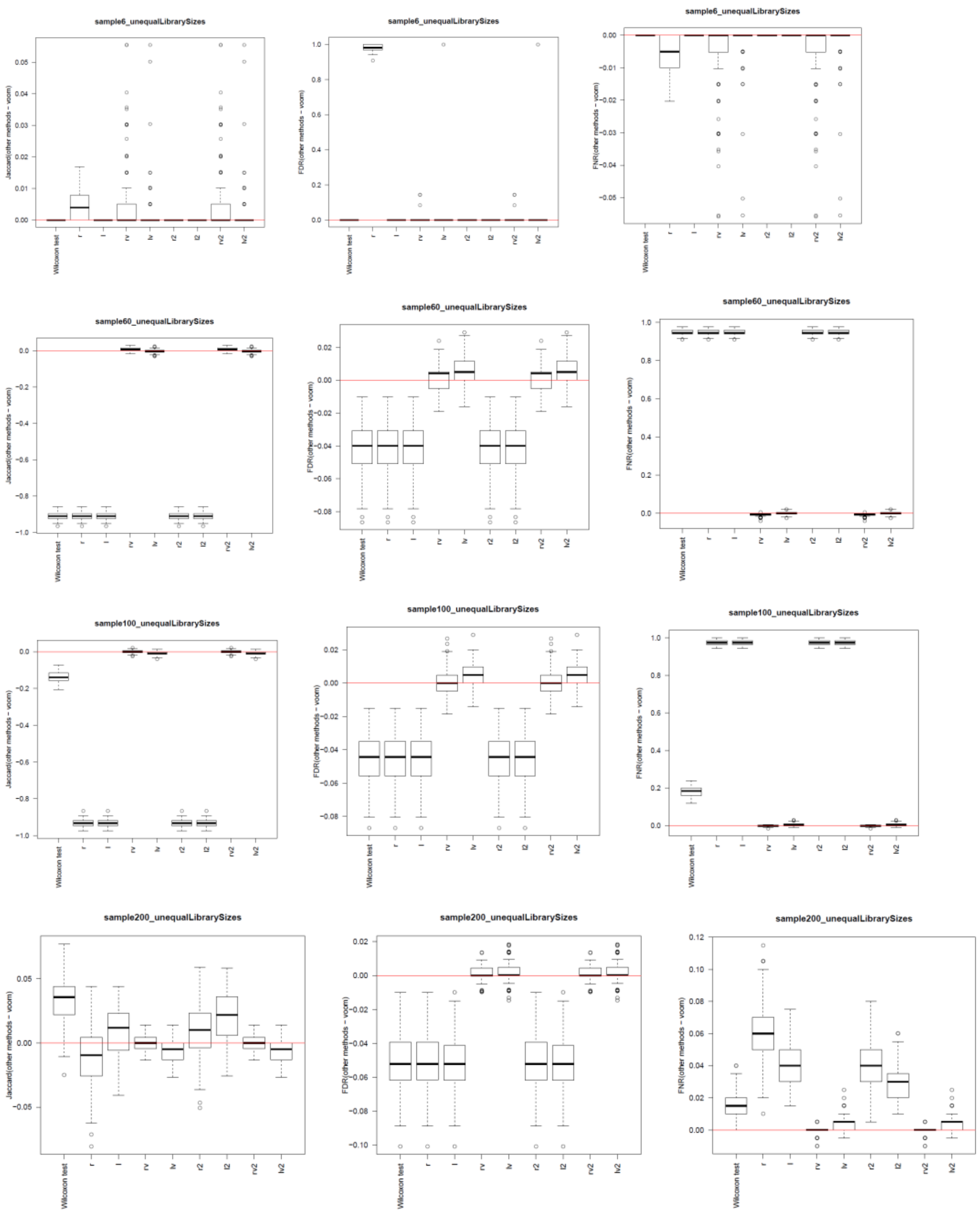

Supplementary Figure 1. The boxplots of the differences of performance indices between voom and other methods (other – voom). The left column is for Jaccard index. Positive values indicate other methods are better than limma with voom transformation. The middle column and right column are for FDR and FNR, respectively. Negative values indicate other methods are better than limma with voom transformation. Top row is for nCases=nControls=3. The second row is for nCases=nControls=30. The 3rd row is for nCases=nControls=50. The bottom row is for nCases=nControls=100.

Supplementary Document

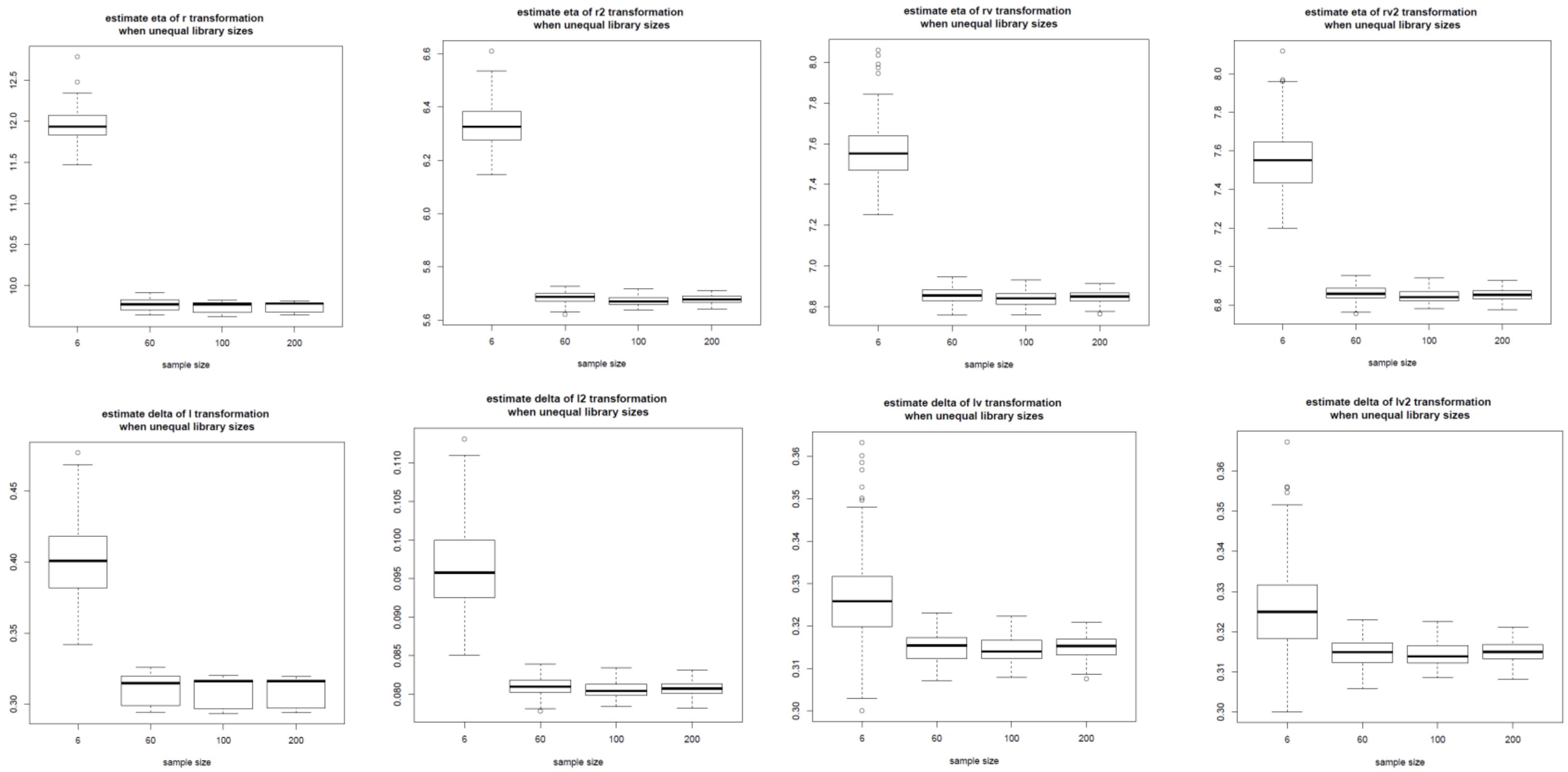

Supplementary Figure 2. Boxplots of the estimated model parameters for the 8 proposed data transformations. The upper panel is for the root transformation. The lower panel is for the log transformation. For each of the 8 proposed data transformation, the estimated parameters drop as sample size increases from 6 to 60, then keep stable.

Supplementary Document

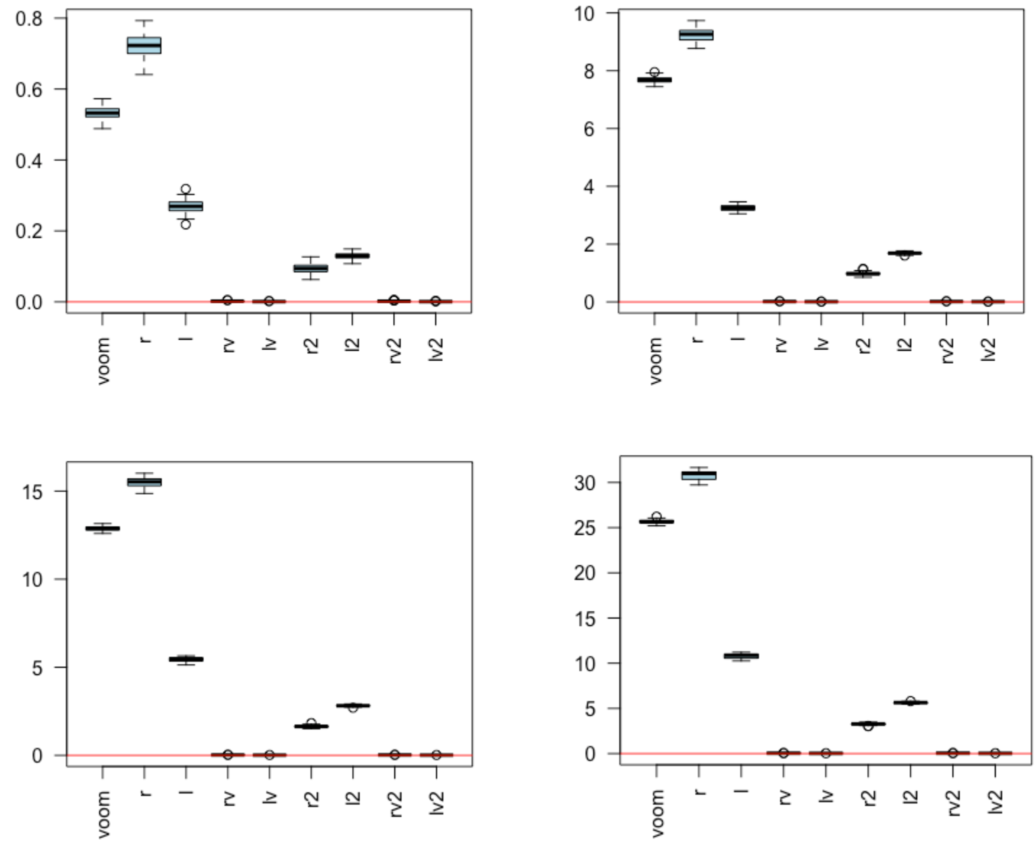

Supplementary Figure 3. The y-axis is the sum of sample-wise squared differences between sample mean and sample median after data transformation in our simulation studies. Top Left panel: nCases=nControls=3.   Top right panel: nCases=nControls=30; Bottom left pane: nCases=nControls=50; Bottom right panel: nCases=nControls=100.

Supplementary Document

Supplementary Table 1. FDR and FNR for SEQC data analyses (nCases=nControls=4)

	Wilcoxon test	voom	r	l	rv	lv	r2	l2	rv2	lv2
FDR	0	0.537037	0.306962	0.3654485	0.4955357	0.5374449	0.306962	0.3654485	0.4955357	0.5374449
FNR	1	0.4230769	0.4384615	0.5102564	0.4205128	0.4615385	0.4384615	0.5102564	0.4205128	0.4615385

Supplementary Document

Supplementary Table 2. FDR and FNR for ERCC (SEQC spike-in) data analyses (nCases=nControls=4)

	Wilcoxon test	voom	r	l	rv	lv	r2	l2	rv2	lv2
FDR	0	0.2	0.2195122	0.1794872	0.1714286	0.1764706	0.2195122	0.1794872	0.1714286	0.1764706
FNR	1	0.5882353	0.5294118	0.5294118	0.5735294	0.5882353	0.5294118	0.5294118	0.5735294	0.5882353
